# Reference-independent comparative metagenomics using cross-assembly: crAss

**DOI:** 10.1093/bioinformatics/bts613

**Published:** 2012-10-16

**Authors:** Bas E. Dutilh, Robert Schmieder, Jim Nulton, Ben Felts, Peter Salamon, Robert A. Edwards, John L. Mokili

**Affiliations:** ^1^Centre for Molecular and Biomolecular Informatics, Nijmegen Centre for Molecular Life Sciences, Radboud University Medical Centre, 6525 GA Nijmegen, The Netherlands, ^2^Department of Computer Science, ^3^Department of Biology, ^4^Computational Science Research Center and ^5^Department of Mathematics, San Diego State University, San Diego, CA 92182, USA and ^6^Division of Mathematics and Computer Science, Argonne National Laboratory, IL 60439, USA

## Abstract

**Motivation:** Metagenomes are often characterized by high levels of unknown sequences. Reads derived from known microorganisms can easily be identified and analyzed using fast homology search algorithms and a suitable reference database, but the unknown sequences are often ignored in further analyses, biasing conclusions. Nevertheless, it is possible to use more data in a comparative metagenomic analysis by creating a cross-assembly of all reads, i.e. a single assembly of reads from different samples. Comparative metagenomics studies the interrelationships between metagenomes from different samples. Using an assembly algorithm is a fast and intuitive way to link (partially) homologous reads without requiring a database of reference sequences.

**Results:** Here, we introduce crAss, a novel bioinformatic tool that enables fast simple analysis of cross-assembly files, yielding distances between all metagenomic sample pairs and an insightful image displaying the similarities.

**Availability and implementation:** crAss is available as a web server at http://edwards.sdsu.edu/crass/, and the Perl source code can be downloaded to run as a stand-alone command line tool.

**Contact:** dutilh@cmbi.ru.nl

**Supplementary information:** Supplementary data are available at *Bioinformatics* online.

## 1 INTRODUCTION

The sequencing of environmental samples has disclosed a universe of novel microbial organisms. Microbiologists and virologists are sequencing DNA and RNA from environments ranging from coral reefs to the human gut and are discovering previously unknown microorganisms in all these samples (Mokili *et al.*, 2012). The fraction of unknown sequences can be particularly high in viral metagenomes because (i) viruses exhibit relatively high mutation rates resulting in distant unrecognizable homology (Bonhoeffer and Sniegowski, 2002), and (ii) this superkingdom has remained poorly explored, with relatively few sequences present in the databases. These properties hinder the comparison of metagenomic samples with high percentages (up to 60–99%) of unknowns (Mokili *et al.*, 2012) by traditional methods that map sequencing reads to known annotated references (Meyer *et al.*, 2008).

In addition, the abundance and diversity of viruses in natural systems impede comparisons between metagenomic datasets, especially when comparing relatively short reads generated by some second-generation sequencing platforms. A promising solution is to initially combine the different metagenomes and perform a *de novo* assembly of all sequence reads. If applied conservatively to avoid chimerization, metagenome assembly can reliably link reads that are completely unknown to reads that have similarity to annotated sequences.

The tool PHAge Communities from Contig Spectra (PHACCS) assesses the biodiversity of uncultured viral communities by mathematically modeling the community structure using the contig spectrum of metagenome assemblies (Angly *et al.*, 2005). PHAge Communities from Contig Spectra was extended to assess cross-assemblies in a tool called Maxiφ (Angly *et al.*, 2006). Because they do not rely on a database with reference sequences, these tools provide an approach for comparing complete metagenomes, even if they contain a high fraction of unknown sequences.

Here, we present crAss, a novel more direct approach based on the same concept, i.e. using cross-assembly of reads from different metagenomes to assess the degree of similarity between the sampled communities. Thus, cross-metagenome assembly enables a sensitive comparison of entities that are shared between samples, including viruses with no homology to known sequences. Unlike previous methods, crAss calculates a pairwise distance score between metagenomes and creates an insightful image to display these distances graphically. Moreover, a cross-assembly combines short sequencing reads into longer contigs that may be more suitable for annotation. For example, downstream analyses could include sensitive homology searches (Soding, 2005), RNA structure detection (Hofacker and Stadler, 2006) and prediction of alternative genetic codes (Dutilh *et al.*, 2011).

## 2 METHODS

### 2.1 General approach

First, the user will combine the reads from all metagenomic datasets to be compared into a cross-assembly, i.e. a single assembly from two or more metagenomes. Note that it is important that all reads have unique identifiers across all the datasets combined. Cross-assembly can be done with a *de novo* assembly tool of choice such as gsAssembler (Margulies *et al.*, 2005), MIRA (Chevreux *et al.*, 2004) or Velvet (Zerbino and Birney, 2008). We note that it is beyond the scope of this article to evaluate the performance of the different assembly tools and all the possible parameter settings (Lin *et al.*, 2011), although it seems that assembler settings optimized for sequence fragments, such as expressed sequence tags (ESTs), may perform better than assembler settings optimized for complete genomes (see Supplementary File 2). The basic idea is that an assembly program will link metagenomic reads derived from the same biological source to one another in a well defined way. The resulting contigs may be interpreted as ‘metagenomic entities’ or traits that are shared between the sampled environments. The results shown later to illustrate crAss were generated using gsAssembler (Margulies *et al.*, 2005) with default parameters, but the user can achieve similar results with alternative assembly tools.

The crAss program takes as input the individual read files (one per metagenome) in Fasta or Fastq format, as well as the ACE file that contains the information about the assembled contigs. We note that the user can strip the potentially privacy-sensitive sequence information from these files before uploading, as is explained on the crAss help page. Examples of input files for crAss analysis, including a simplified toy example, are available through the crAss web site (http://edwards.sdsu.edu/crass). For every contig, crAss counts the number of reads derived from each metagenome and determines whether the contig is shared between two or more samples (cross-contig). The degree of relatedness between two metagenomes is calculated from this information according to four distance formulas (see later).

The output of crAss consists of (i) a list of all contigs showing how many reads from each metagenome it is composed of (for reference, this list includes the identifiers of the unassembled singleton reads, which are not used in the distance calculations), (ii) a symmetrical distance matrix for each distance formula that contains the distances between all pairs of metagenomes, and (iii) an image that summarizes the similarities between the metagenomes (see Section 3). If the user combined two or three metagenomes, the image will be an XY-plot or an XYZ-plot, respectively, visualized using Gnuplot. 3D analyses are also presented as a triangle plot. Note that a user can use the data in the contig list to create other visualizations. If the user combined more than three metagenomes, there will be one image for every distance formula, showing a cladogram created on the basis of the respective distance matrix using BioNJ (Gascuel, 1997), and visualized using Drawtree version 3.68 from the Phylip package (Felsenstein, 1989).

### 2.2 Distance formulas

Comparison tools like Maxiφ (Angly *et al.*, 2006) parametrize the deformation necessary to morph one metagenome into another. crAss uses a single dimension, a distance, for the comparisons. There are always many ways to define distances between given sets of numbers. In crAss, the distance between all pairs of metagenomes is calculated using four possible distance formulas. The first is Equation (1), a formula that has been used to correct for genome size when calculating phylogenetic distances between species (Dutilh *et al.*, 2004; Dutilh *et al.*, 2007; Korbel *et al.*, 2002). It is used here to correct for metagenome size when calculating distances between samples.

**Equation 1.** Distance formula ‘SHOT’ (Korbel *et al.*, 2002) used for calculating the distance *d_i,j_* between metagenomes *i* and *j* based on the number of contigs *c_i_* and *c_j_* that contain reads from these metagenomes, respectively, and the number of cross-contigs *c_i,j_* with reads from both metagenomes.

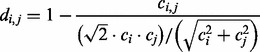



The idea of this formula is to normalize the similarity signal *c_i,j_* for metagenome size by dividing by an appropriate mean value of *c_i_* and *c_j_*, in this case the square of the geometric mean divided by the root mean square. As we will see, this mean is never far from the minimum of *c_i_* and *c_j_*, *min*{*c_i_,c_j_*}. Therefore, when *c_i_* and *c_j_* are far apart in value, the signal *c_i,j_* is not attenuated by dividing by a larger number than necessary. It is instructive to rewrite the second term in *Equation (1)* as follows [compare with Equation (4)]:

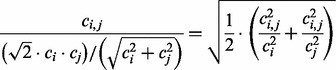



Here, the similarity measure is seen as the root mean square of the separately normalized quantities *c_i,j_*/*c_i_* and *c_i,j_*/*c_j_*. In this form, it is easy to see that we have the following inequality:

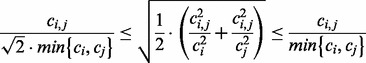



This points to the second distance metric, where we normalize the similarity signal *c_i,j_* for metagenome size by dividing by the smaller of the two metagenomes.

**Equation 2.** Distance formula ‘minimum’ used for calculating the distance *d_i,j_* between metagenomes *i* and *j* based on the number of contigs *c_i_* and *c_j_* that contain reads from these metagenomes, respectively, and the number of cross-contigs *c_i,j_* with reads from both metagenomes.

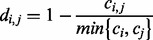



Both Equations (1) and (2) are presence/absence-based formulas. They assume that the presence of a contig or metagenomic trait in a sample (i.e. the contig contains at least one read from that sample) is more informative than its abundance. In our experience, such a qualitative comparison can give insightful information for comparing metagenomes because the number of reads assembled into a contig may not always reflect a truly random sample from the community metagenome, owing to biases that may result at the sampling, sequencing or assembly level. However, we also include two more qualitative distance measures, where the number of reads is assumed to reflect the abundance of the contig in the environment. In the first, we index the complete set of observed contigs by *k* = 1,…,*n*, and let *r_ki_* denote the number of reads in contig *k* from metagenome *i*. We assume that the fraction of reads from metagenome *i* that are incorporated into contig *k.*

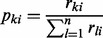

is a measure of the relative importance of contig *k* to metagenome *i*, and that the probability vector (or distribution)

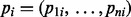

characterizes the metagenome *i*. Wootters has proposed a natural statistical measure of distance between probability distributions (Wootters, 1981) that is closely related to distance measures associated with the names of Fisher and Amari (Amari, 1982). It is based generally on the minimum number of jumps that is required to get from one distribution to another, where a jump is a statistical fluctuation typical of a finite sample from the distribution. The distance is normalized in such a way that it is independent of the sample size used to establish a statistical fluctuation. This Wootters metric can be adapted to reflect the distance between metagenomes as follows.

**Equation 3.** Distance formula ‘Wootters’ (Wootters, 1981) used for calculating the distance *d_i,j_* between metagenomes *i* and *j* based on the fraction of reads *p_ki_* and *p_kj_* from these metagenomes, respectively, that are incorporated into contig *k*.

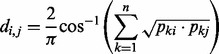



As it may be expected that longer contigs attract more reads from unrelated metagenomes by chance, we include a fourth distance formula that mirrors Equation (1), but is based not on the fraction of cross-contigs but on the fraction of reads assembled into cross-contigs. Like Equation (3), this is a quantitative comparison that accounts for skewed read distributions in contigs and corrects for biases, e.g. owing to differences in contig length.

**Equation 4.** Distance formula ‘reads’ used for calculating the distance *d_i,j_* between metagenomes *i* and *j* based on the number of reads *r_i_* and *r_j_* from these metagenomes, respectively, that are incorporated into contigs, and the number of reads *r_i,__j_* and *r_j__,__i_* from these metagenomes, respectively, that are incorporated into shared cross-contigs.

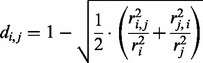



Each of the four distance formulas that crAss offers is mathematically natural for the structure of the data. Our expectation is that each will prove useful for revealing interrelationships of particular sorts. Just which metric will be most suitable for which type of comparison will teach us about the structure of these relationships. We invite users to share their experience on the SEQanswers user forum (Li *et al.*, 2012), available through the crAss web site, and let future users know which distance formula they found useful or useless for their particular application.

### 2.3 Real data

To illustrate the use of crAss for reference-independent comparative metagenomics, we selected three sets of viral metagenomes sequenced using the GS FLX system (Lopez-Bueno *et al.*, 2009; Nakamura *et al.*, 2009; Rosario *et al.*, 2009). We used viral metagenomes because they are more challenging than microbial metagenomes because of the high diversity and large fraction of unknown sequences. Table 1 shows the 15 samples from these publications that we used as test data for crAss. All reads were preprocessed using a pipeline including cross_match (http://www.phrap.org/) to remove possible vector contamination, TagCleaner (Schmieder *et al.*, 2010) to remove sequencing tags, PRINSEQ (Schmieder and Edwards, 2011b) to filter low quality, short and duplicate sequences and DeconSeq (Schmieder and Edwards, 2011a) to remove human-like sequence contamination. The details of the preprocessing pipeline are available at http://edwards.sdsu.edu/mymgdb/. We then combined the 15 datasets into a single cross-assembly using gsAssembler 2.6 (Margulies *et al.*, 2005) with default parameters. Finally, we uploaded the individual raw reads files, as well as the ACE file resulting from the assembly, to the crAss web server to produce the presented results.

**Table 1.** Viral metagenomic datasets used to illustrate the use of crAss for reference-independent comparative metagenomics

Dataset	Reference
Antarctic spring lake viral metagenome	(Lopez-Bueno *et al.*, 2009)
Antarctic summer lake viral metagenome	
Human fecal sample N1 viral metagenome	(Nakamura *et al.*, 2009)
Human fecal sample N2 viral metagenome	
Human fecal sample N3 viral metagenome	
Human fecal sample N4 viral metagenome	
Human fecal sample N5 viral metagenome	
Human nasal sample F1 viral metagenome	
Human nasal sample F2 viral metagenome	
Human nasal sample F3 viral metagenome	
Reclaimed effluent freshwater DNA viral metagenome	(Rosario *et al.*, 2009)
Reclaimed effluent freshwater RNA viral metagenome	
Reclaimed Nursery freshwater DNA viral metagenome	
Reclaimed nursery freshwater RNA viral metagenome	
Potable freshwater DNA viral metagenome	

### 2.4 Simulated data

We tested the performance of the different distance formulas in crAss using simulated metagenomic samples created from completely sequenced bacterial genomes. Genomes between 2 and 6 Mb in length representing the phyla *Actinobacteria* (*n* = 77), *Firmicutes* (*n* = 154) and *Proteobacteria* (*n* = 280) were selected from RefSeq (Pruitt *et al.*, 2012), choosing one genome per species to avoid redundancy (Supplementary File 1). We then created several sets of simulated metagenomes using Grinder 0.4.5 (Angly *et al.*, 2012) with realistic pyrosequencing parameters: read length = 450 ± 100 nt, and 2% sequencing errors, of which 85% were substitutions and 15% were indels; homopolymeric errors were included according to the Balzer model (Balzer *et al.*, 2011).

Three experiments were carried out (Supplementary File 1). First, we investigated the effect of decreasing species overlap by creating 32 simulated metagenomes of 30 species each. Samples ov00 and ov01 contained the same thirty *Firmicutes*, and in every subsequent sample (ov02–ov31), we randomly replaced one of the *Firmicutes* by a *Proteobacterium*. Each sample was cross-assembled with ov00 to determine how the similarity score depends on the species overlap.

Second, we investigated the effect of varying species abundance. We created ten simulated metagenomes containing the same 10 *Firmicutes* genomes, but with different logarithmic species distributions (in five pairs, see Supplementary File 1). These 10 samples were cross-assembled together.

Third, we investigated the sensitivity for detecting similar (but not the same) species (either *Actinobacteria* or *Firmicutes*) against a background of noise from a third phylum (*Proteobacteria*). We created 11 sets of samples, each consisting of nine metagenomes. Three of those contained 25 or 26 randomly selected (but different) *Actinobacteria*, and six contained 25 or 26 *Firmicutes*. Within these metagenomes, the *Actinobacteria* or *Firmicutes* ‘signal’ was contaminated with an increasing percentage of *Proteobacteria* ‘noise’, sampled uniformly from the 280 genomes. The species distributions in all these samples are described in Supplementary File 1, and the results from these experiments are presented in Supplementary File 2.

### 2.5 Short *k*-mer profiles

Short *k*-mer profiles have previously been used as an alternative approach for reference-independent comparative metagenomics, where a length of *k* = 2 was shown to yield the best separation (Willner *et al.*, 2009). Distances *d_i,j_* between metagenomes *i* and *j* were calculated from *k*-mer profiles as:

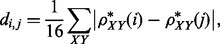

where *XY* are the dinucleotides, and the dinucleotide odds ratio 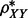 is calculated from the corrected frequencies 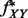 (forward and reverse averaged) as follows (Karlin and Mrazek, 1997; Willner *et al.*, 2009):

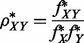



### 2.6 Reference mapping

We compared the performance of crAss with a reference mapping analysis. All reads were queried by blastn 2.2.25+ (Camacho *et al.*, 2009) (default parameters, *E*-value ≤0.001) to the Genbank non-redundant nucleotide database (nt, October 19th, 2011) and assigned to the taxonomic clade of their closest hit. Read counts were divided in case of draws and iteratively summed for all parent clades as explained previously (Trindade-Silva *et al.*, 2012). Distances between datasets were calculated as one minus the correlation coefficient of the resulting taxonomic distributions.

## 3 RESULTS

It is expected that metagenomes from a similar environment share a larger fraction of microbial or viral species than metagenomes from different origins. These shared ‘metagenomic entities’ can be reconstructed by cross-assembly of the metagenomes and analyzed and visualized using crAss. The contigs that link two samples (cross-contigs) directly represent the similarity between those samples. If most contigs are composed of a mix of reads from different samples, then it may be concluded that there are many shared entities among those samples. Conversely, if contigs are predominantly composed of reads from either one of the samples, but there are few cross-contigs, these samples are highly dissimilar in composition.

### 3.1 Simulation experiments

To evaluate the performance of each of the distance formulas and compare cross-assembly with existing approached for comparative metagenomics, we created several simulated metagenomes by sampling reads from completely sequenced bacterial genomes (Supplementary File 1).

**Fig. 1.**
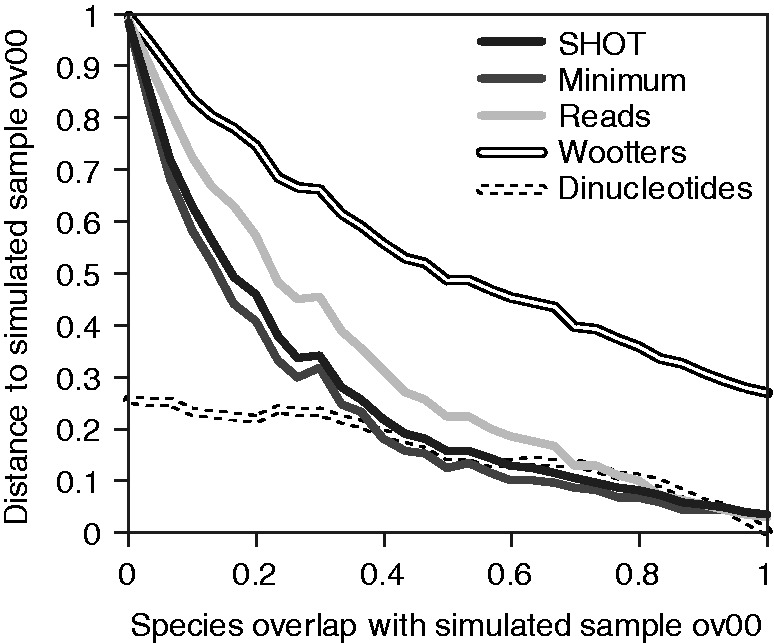
Distance between 31 simulated metagenomic samples with increasing species overlap, and simulated sample ov00 (see Supplementary File 1 for species distributions). Distances were calculated using the four crAss distance formulas; the fifth line shows the distance based on dinucleotide odds ratios (Willner *et al.*, 2009). See Section 2 for details

First, we investigated a sequence of samples with decreasing species overlap by creating 31 simulated datasets of 30 species each (ov01–ov31); the first contained 30 *Firmicutes*, and one of those was replaced by a *Proteobacterium* in each subsequent dataset. Each of these was cross-assembled with another dataset of 30 *Firmicutes* (ov00, which was sampled from the same species as ov01, see Supplementary File 1). As shown in Figure 1, the distance to sample ov00 decreases with increasing species overlap for all distance formulas. As a result of the assembly parameters in Newbler, which are optimized for complete genome assembly, the curves show a non-linear response of the distance score to the degree of species overlap. For comparison, the second tab in Supplementary File 2 shows MIRA (Chevreux *et al.*, 2004) assemblies optimized for either complete genomes (tries to build long contigs) or ESTs (allows shorter fragments), which shows a more linear response. Indeed, the EST parameters might be more appropriate for metagenome assembly as well.

Second, we investigated the effect of varying species abundance by creating five pairs of simulated metagenomic samples with different logarithmically distributed species abundances (see Supplementary File 1 for species distributions). As shown in Supplementary Figure S1, all distance formulas cluster the correct sample pairs together. Interestingly, the quantitative distance formula ‘Wootters’ [Equation (3)], as well as the dinucleotide odds ratios (Willner *et al.*, 2009), captures the connection between the clusters (ab03, ab04) and (ab05, ab06), which both have the highest fraction of *Bacillus weihenstephanensis* KBAB4 reads (70%, see Supplementary File 1), and subsequently cluster (ab07, ab08), with 21% *B.weihenstephanensis* KBAB4 reads. In contrast, the qualitative distance formulas ‘SHOT’ [Equation (1)] and ‘minimum’ [Equation (2)] do not capture these connections, nor does the ‘reads’ formula [Equation (4)].

**Fig. 2.**
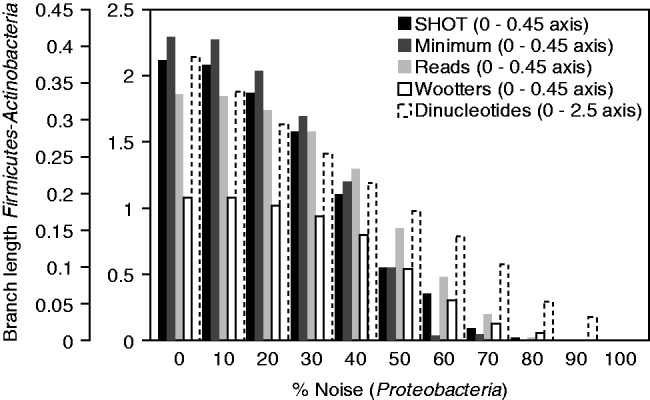
In cladograms of nine simulated metagenomes, those containing *Actinobacteria* (*n* = 3) mostly form a separate cluster from those containing *Firmicutes* (*n* = 6; Supplementary Fig. S2). The length of the separating internal branch is plotted with increasing *Proteobacteria* contamination, until the *Actinobacteria* and *Firmicutes* metagenomes no longer form separate clusters, and there is no internal branch. See text for details. The cladograms created by crAss are available in Supplementary Figure S2

Third, we evaluated the sensitivity of crAss for detecting different species from the same phylum in the face of increasing levels of noise. Simulated metagenomic samples were created from the complete genomes of 511 species (see Supplementary File 1 for species distributions). Three metagenomes contained 25 or 26 different *Actinobacteria* each, and six metagenomes contained 25 or 26 different *Firmicutes* each. These nine datasets were contaminated with 0–100% noise in the form of simulated reads from *Proteobacteria* genomes. The results of this analysis show that crAss can separate the *Actinobacteria* from the *Firmicutes* metagenomes, with up to 80% *Proteobacteria* contamination (70% for the distance formula ‘minimum’, see Fig. 2). As expected, the approach based on dinucleotide odds ratios also separates *Firmicutes* (also known as low G + C gram-positive bacteria) from *Actinobacteria* (or high G + C gram-positive bacteria), even when contaminated with up to a 90% *Proteobacteria* reads.

To summarize, crAss clusters metagenomes containing different species from the same phylum in the face of high levels of noise derived from identical contaminating species sets. Moreover, the length of the branch that separates these groups decreases with the decrease in signal for all distance formulas (Fig. 2), showing that crAss can sensitively identify the degree of similarity between samples.

### 3.2 Comparing publically available viral metagenomes

**Fig. 3.**
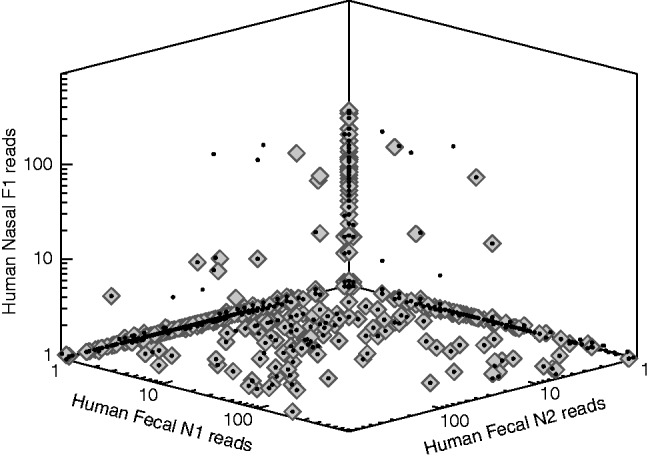
Cross-assembled metagenomic reads from one human nasal sample and two human fecal samples. Each gray diamond represents a contig. The X, Y and Z coordinates indicate the number of incorporated reads from the metagenomes mentioned along the axes. Note that zero values are set to 0.9 so they can be displayed on the logarithmic plot. Small black dots are the projections of the diamonds onto the planes, but superimposed for visibility. A triangle plot of the same data is also available. These graphs can be retrieved at http://edwards.sdsu.edu/crass/ under Job ID 1329506771

crAss visualizes the similarity between metagenomes in different ways depending on the number of metagenomes combined in the cross-assembly. Two or three samples will be displayed as an XY-plot or an XYZ-plot, respectively, showing the number of reads combined into each of the contigs. 3D analyses are also presented as a triangle plot. Figure 3 displays an illustrative example of a cross-assembly between three metagenomes, showing shared and non-shared contigs. Contigs composed solely of reads from a single metagenome will be plotted along the axes, as only one metagenome will have a non-zero value. In the example shown in Figure 3, there are 76 contigs that contain only reads from the fecal N1 sample, 338 contigs with only fecal N2 reads and 152 contigs with only nasal F1 reads. Furthermore, this plot shows that 176 of the 230 cross-contigs lie in the bottom plane, indicating that they contain reads from both the fecal samples but not from the nasal sample. Only 24 contigs contain reads from all three metagenomes. These details are available in the crAss output file output.contigs2reads.txt, which can be downloaded, e.g. for alternative visualization in a program of choice. The results illustrate, as expected, that the fecal samples are more similar to one another than either of them are to the nasal sample.

More than three metagenomes cannot be easily visualized in such a plot, so when more than three datasets are used, crAss outputs a cladogram that shows the similarities between samples. The cladogram is calculated from the distance matrix using BioNJ (Gascuel, 1997), and crAss outputs a different one for each of the four distance formulas. Moreover, we present two versions of each cladogram, a version with branch lengths that represent the distances between the samples and one where the branch lengths are ignored. This may be useful if the differences between metagenomes are much more pronounced than the similarities, leading to short internal branch lengths.

**Fig. 4.**
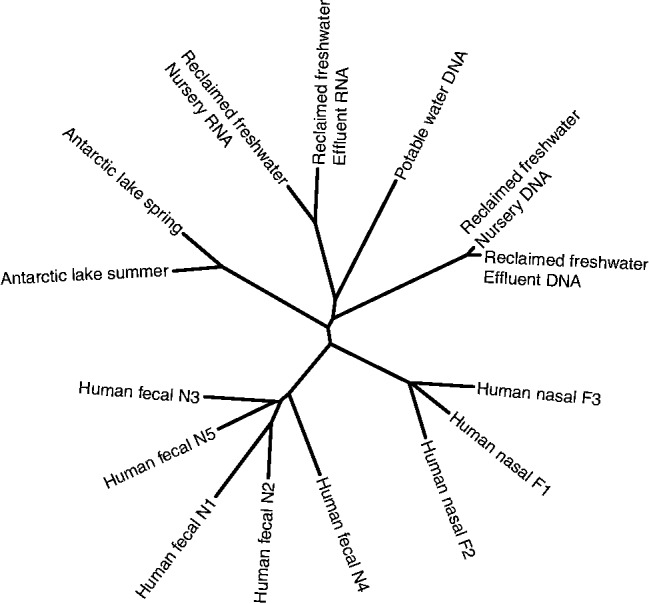
Cladogram representing the distance between metagenomes based on the fraction of cross-assembled contigs between all sample pairs. crAss creates this cladogram from a distance matrix using BioNJ (Gascuel, 1997) and visualizes it using Drawtree (Felsenstein, 1989). This cladogram was based on Equation (1). The complete output for this dataset, including distance matrices and cladograms based on the other distance formulas, can be retrieved at http://edwards.sdsu.edu/crass/ under Job ID 1329505996

The latter cross-assembly uses 53.1 ± 26.6% of the reads from the 15 metagenomes for the comparison. A comparative metagenomic analysis based on blastn mapping of the reads to the Genbank reference database uses 45.9 ± 43.1% of the reads, slightly less and with a much wider spread because of the difference in the eight human versus seven water samples (84.3 ± 9.5% and 1.9 ± 1.7% reads mapped, respectively, see Supplementary Fig. S3A). Human-associated microbes have traditionally been abundant in the databases, whereas microbes from other biomes remain underrepresented. Although a cladogram based on the correlation coefficient of the taxonomic distributions of these hits (see Section 2) shows some clustering of biomes (Supplementary Fig. S3B), it is not as accurate as the crAss analysis in Figure 4. Water samples occur in separate clusters, and the human nasal samples are separated from human fecal samples. We suspect that this is a long-branch attraction artifact, caused by the water samples, which are unlike any of the other samples, being pushed to the center of the cladogram.

## 4 DISCUSSION

Determining the interrelationships between metagenomes from different biomes or different time points is important to understand the microbial world around us. Mapping metagenomic sequences to a reference database of known genes is a feasible approach to transfer taxonomical and functional annotations to sequence reads. However, it can limit the amount of data that can be analyzed because the majority of the sequencing reads in difficult-to-annotate datasets, such as viral metagenomes from biomes other than the human microbiome, lack known homologs (Mokili *et al.*, 2012). A promising alternative is reference-independent comparative metagenomics.

We present crAss, an intuitive approach for comparative metagenomics that calculates a similarity signal using shared entities, which are identified by cross-assembly. The tool does not depend on homology of the sequenced reads to any known sequence in a reference database, or even on full-length homology of the reads to one another. Assembly algorithms, such as those used to create the ACE files required for crAss analysis, identify shared subsequences (long or signature *k*-mers) between reads to assemble them (Li *et al.*, 2011). Because it relies on a different similarity signal, this long *k*-mer approach is complementary to the use of short *k*-mer profiles (Ghosh *et al.*, 2011; Willner *et al.*, 2009).

We expect that Equations (1) and (2) can be applied for qualitative comparisons between environments because they only take the degree of shared entities (cross-contigs) into account. Conversely, Equations (3) and (4) will be more suited for quantitative comparisons because they consider the read counts as a measure of abundance. Although limited in scope, our current results based on analyses of simulated metagenomic samples suggest that the distance formula ‘Wootters’ [Equation (3)] may outperform the other formulas for detecting both qualitative and quantitative signals, with similar performance as the comparisons based on dinucleotide odds ratios (Willner *et al.*, 2009). Nevertheless, it is difficult to predict which of the distance formulas presented in this article will be the most suitable for which biological application. That is why we have created a user forum at SEQanswers (Li *et al.*, 2012), available through the crAss web site, where we hope that users will share their experiences and any publications that use crAss.

An additional advantage of using cross-assembly is that the sequences of biological entities, which are shared between specific samples, are simultaneously assembled. Based on the results obtained using simulated data (Supplementary File 2), we expect that fragment assemblers developed for ESTs, such as MIRA, or specialized metagenome assemblers will be more suitable for cross-metagenome assembly than assemblers like Newbler, which are optimized for single-genome assembly.

If the parameters of the assembly program are set to strict values to avoid chimerization, tentative taxonomic annotations may be transferred between reads that are linked within one contig. We recommend using parameter settings that the user is comfortable with for metagenomic assembly, but it should be noted that the resulting crAss cladogram is based on a similarity score between entire metagenomes, which limits the effect of possibly rare chimerical sequences. Moreover, chimerization may be problematic between closely related species, but less severe for more distant organisms. Thus, although they will add some noise to the similarity signal, chimeras are more likely to link reads from biomes that are already similar, alleviating errors in the final cladogram. To address the issue of potential chimerization, we recommend creating separate cross-assemblies and crAss cladograms with stringent and permissive assembly parameters, which will lead to fewer and more chimerical contigs, respectively. The resulting cladograms can then be compared to evaluate the effect of chimerization on the cross-assembly analysis. In general, we have seen that the cladogram rarely changes with altered assembly parameters, indicating that crAss is a robust approach for comparative metagenomics.

*Funding*: NSF Division of Biological Infrastructure grant 0850356 and Division of Environmental Biology grant 1046413 (to R.A.E.). Dutch Science foundation (NWO) Veni grant 016.111.075 (to B.E.D.).

*Conflict of Interest*: none declared.

## Supplementary Material

Supplementary Data
