# Supplementary material for: Reference-independent comparative metagenomics using cross-assembly: crAss
Source: Bioinformatics. 2012 Oct 16;28(24):3225–31. doi: 10.1093/bioinformatics/bts613 (PMC3519457; doi:10.1093/bioinformatics/bts613)
Supplement: Supplementary Data [file supp_28_24_3225__index.html]

Reference-independent comparative metagenomics using cross-assembly: crAss — Reference-independent comparative metagenomics using cross-assembly: crAss — Supplementary Data 

# Reference-independent comparative metagenomics using cross-assembly: crAss

## Supplementary Data

files

**Files in this Data Supplement:**

- Supplementary Data - eps file
- Supplementary Data - eps file
- Supplementary Data - eps file
- Supplementary Data - xlsx file
- Supplementary Data - xlsx file
